# Novel dual-targeting c-Myc inhibitor D347-2761 represses myeloma growth via blocking c-Myc/Max heterodimerization and disturbing its stability

**DOI:** 10.1186/s12964-022-00868-6

**Published:** 2022-05-26

**Authors:** Ruosi Yao, Menghui Zhang, Jian Zhou, Linlin Liu, Yan Zhang, Jian Gao, Kailin Xu

**Affiliations:** 1 Blood Diseases Institute, Xuzhou Medical University, Xuzhou, Jiangsu China; 2 Department of Hematology, The Affiliated Hospital of Xuzhou Medical University, Xuzhou, Jiangsu China; 3 College of Medical Imaging, Xuzhou Medical University, Xuzhou, Jiangsu China; 4 Jiangsu Key Laboratory of New Drug Research and Clinical Pharmacy, Xuzhou Medical University, Xuzhou, Jiangsu China; 5 Xuzhou Ruihu Health Management and Consulting Co., Ltd, Xuzhou, Jiangsu China

**Keywords:** c-Myc inhibitor, Multiple myeloma, DNA damage, Drug resistance, Apoptosis

## Abstract

**Background:**

Transcription factor c-Myc plays a critical role in various physiological and pathological events. c-Myc gene rearrangement is closely associated with multiple myeloma (MM) progression and drug resistance. Thereby, targeting c-Myc is expected to be a useful therapeutic strategy for hematological disease, especially in MM.

**Methods:**

Molecular docking-based virtual screening and dual-luciferase reporter gene assay were used to identify novel c-Myc inhibitors. Cell viability and flow cytometry were performed for evaluating myeloma cytotoxicity. Western blot, immunofluorescence, immunoprecipitation, GST pull down and Electrophoretic Mobility Shift Assay were performed for protein expression and interaction between c-Myc and Max. c-Myc downstream targets were measured by Q-PCR and Chromatin immunoprecipitation methods. Animal experiments were used to detect myeloma xenograft and infiltration in vivo.

**Results:**

We successfully identified a novel c-Myc inhibitor D347-2761, which hindered the formation of c-Myc/Max heterodimer and disturbed c-Myc protein stability simultaneously. Compound D347-2761 dose-and time-dependently inhibited myeloma cell proliferation and induced apoptosis. Dual knockout Bak/Bax partially restored D347-2761-mediated cell death. Additionally, compound D347-2761 could, in combination with bortezomib (BTZ), enhance MM cell DNA damage and overcome BTZ drug resistance. Our in vivo studies also showed that compound D347-2761 repressed myeloma growth and distal infiltration by downregulating c-Myc expression. Mechanistically, novel dual-targeting c-Myc inhibitor D347-2761 promoted c-Myc protein degradation via stimulating c-Myc Thr58 phosphorylation levels, which ultimately led to transcriptional repression of CDK4 promoter activity.

**Conclusions:**

We identified a novel dual-targeting c-Myc small molecular inhibitor D347-2761. And this study may provide a solid foundation for developing a novel therapeutic agent targeting c-Myc.

**Video Abstract**

**Supplementary Information:**

The online version contains supplementary material available at 10.1186/s12964-022-00868-6.

## Background

The transcription factor c-Myc is a member of the basic helix-loop-helix leucine zipper (bHLH-LZ) family, which tends to be highly expressed in many solid tumors and hematological malignancies [1–3]. As a master regulator, c-Myc regulates approximately 15% of genes in the genome and plays a critical role in various biological processes, including cell cycle, apoptosis, differentiation, and glucose metabolism [4, 5]. Multiple myeloma (MM) is one of the most common hematological tumors, which is still incurable. Although great significant advances have been made in diagnosis and therapy, relapse remains a big problem because of targeting drug resistance. Currently, increasing evidence indicates that c-Myc is a primary oncogene in MM [6]. GSEA analysis demonstrated that c-Myc activation was expressed in approximately 67% of MM patients but not in MGUS plasma cells, implying that a c-Myc activation is an important event in MM disease progression [7]. Other works also showed that targeting c-Myc inhibitor 10058-F4 and JQ1 inhibited MM cells and primary tumor cells' survival and induced apoptosis depending on c-Myc expression [8, 9]. Despite its extreme importance, exploring more effective and low-toxic compounds targeting c-Myc remain challenging.

Silencing c-Myc expression contributed to disturb c-Myc-mediated tumor development and progression. Usually, c-Myc is an unstable transcription factor, which half-life is about 30 min. However, c-Myc stability could be enhanced due to oncogenic mutations affecting c-Myc proteasome degradation in many malignancies [10]. Proteasome-mediated protein degradation is a crucial pathway that controls c-Myc protein function in some biological processes and tumor progression. As RING finger E3 ubiquitin ligases, Fbw7, Skp2, and β-Trcp have been reported to be responsible for c-Myc degradation [11–13]. Previous studies also indicated that Ser62 and Thr58 phosphorylation levels’ status change was closely associated with c-Myc protein stability. High phosphorylation of Ser62 mediated by PIM1 and CAMKIIγ increased c-Myc protein stability, further enhancing c-Myc transcriptional activity, whereas high phosphorylation at Thr58 mediated by Gsk3β was required for c-Myc protein degradation [14–16]. Recently, Noriyuki et al. [17] reported that a polyamine regulatory protein AZ2 interacted with c-Myc to promote its degradation independent of c-Myc ubiquitination or Thr58 phosphorylation. Our previous studies have identified two small molecular inhibitors, 7594-0035 and 7594-0037, based on the c-Myc protein unstable domain, damaging c-Myc protein stability and inducing MM cells apoptosis [18, 19]. Nevertheless, its anti-myeloma activities were poor, and the specific mechanism of action was still unclear.

It has been confirmed that c-Myc usually formed a heterodimer with transcription factor Max to elicit its carcinogenesis. Then the c-Myc/Max heterodimers bound to conserved c-Myc response element E-box motif (CACGTG) to further transcriptional activate or repress downstream target genes [20]. Because it is essential in c-Myc-mediated oncogenesis, targeting c-Myc/Max interaction could be an attractive approach for repressing the c-Myc function. Many small molecular inhibitors were identified based on this strategy, including 10058-F4, 10074-A4, 10074-G5, L755507, MYCi361, sAJM589 [21–23], which showed antitumor activities in varying degrees. In this study, we attempted to screen and identify dual-targeting c-Myc small molecular compounds based on the c-Myc/Max interaction domain via molecular docking-based virtual screening. Herein, we found compound D347-2761 showed thriving anti-MM activities in vitro and in vivo. Meanwhile, compound D347-2761 also induced DNA damage in combination with bortezomib and exhibited a synergistic effect with bortezomib. Mechanistically, compound D347-2761 could disturb c-Myc stability and destroy c-Myc/Max interaction to repress c-Myc downstream target gene CDK4 further. Taken together, we identified a novel dual-targeting c-Myc small molecular inhibitor D347-2761, which could be used against multiple myeloma.

## Materials and methods

### Molecular docking-based virtual screening

The crystal structure of c-Myc/Max complex (PDB ID: 1NKP [24]) was used for the molecular docking-based virtual screening to discover potent c-Myc inhibitors, and ChemDiv database (commercially available and the purity of compounds are greater than 95%) of TopScience Co., Ltd. (Shanghai, China) was selected as a screening library. Since only 2D structural information is available, the compounds in the ChemDiv database were preprocessed by the *dbtranslate* module in Sybyl-X2.1 (Sybyl-X2.1 is available from Tripos Associates Inc., S Hanley Rd., St. Louis, MO 631444, United States). Firstly, Lipinski’s rule of five (Ro5) [25] was conducted on the ChemDiv database to eliminate non-drug-like molecules. Then the well-defined binding pocket, which is formed by the residues Leu917, Phe921, and Lys939 in c-Myc and Arg212, Arg215, Asp216, Ile218, Lys219, Phe222, and Arg239 in Max, was used for the binding site of c-Myc inhibitors as described in the previous study [26]. The missing hydrogen atoms of proteins were added by the biopolymer module during receptor preparation, and all water molecules were removed. To accelerate the virtual screening, a high-speed screening [27] was carried out by decreasing the maximum quantity of conformations and rotatable bonds from 20 to 10 and from 100 to 50, respectively. Then, the molecules with docking scores within the top 1% were normatively screened again using the default docking parameters, which would bring out the top 500 molecules. Finally, eight compounds were selected for commercial purchase by ranking the docking scores and clustering analysis, followed by the dual-luciferase reporter gene assay.

### Cell culture and reagents

Human myeloma cells, RPMI-8226 and NCI-H929 were cultured in RPMI-1640 medium (KeyGEN BioTECH, China) supplemented with 10% FBS (ZETA LIFE, USA) at 37℃ under 5% CO_2_. RPMI-8226/BTZ100 was kindly provided by Dr. Jacqueline Cloos (VU University Medical Center, The Netherlands). Compounds 8019-1964, D125-2215, D347-2649, C630-0056, D347-2563, D011-0486, C630-0144 and D347-2761 were purchased from TopScience Co., Ltd. (Shanghai, China). Commercial c-Myc positive inhibitor 10074-G5 was purchased from MCE (Shanghai, China).

### Cell viability and flow cytometry

Cell viability was measured with a CCK8 kit (KeyGEN BioTECH, China). Briefly, 1 × 10^4^ indicated cells were seeded in 96-well plates and incubated with D347-2761 at different concentrations for a certain time. Then cells were incubated with 5 μl CCK8 for another 2 h, and the absorbance was measured at 450 nm. Apoptotic cells were stained by Annexin V-APC/7-AAD apoptosis kit (KeyGEN BioTECH, China) and subsequently quantified by flow cytometer (BD Biosciences). For cell cycle assay, cells treated by D347-2761 were permeabilized and treated by RNase A, then cells were stained by PI and analyzed by flow cytometry.

### Western blot (WB) and immunofluorescence (IF)

Briefly, cells treated or untreated by D347-2761 were harvested and lysed in RIPA lysis buffer (Beyotime Biotechnology, Shanghai, China). The whole proteins were separated with 10% SDS-PAGE and transferred to the PVDF membrane. Subsequently, the membranes were blocked and incubated with primary antibodies and HRP-conjugated second antibodies, respectively. The following primary antibodies were used: CDK2, Cyclin A, Bax, β-actin, c-Myc, Max and GSK3-β (Proteintech), Bak and γH2AX (ABclonal), p-GSK3-β, p-c-Myc Thr58 and p-c-Myc Ser62 (Affinity). For the IF assay, the treated myeloma cells were washed, fixed, and permeabilized. The anti-γH2AX antibody (ABclonal) was used to detect γH2AX protein location. Finally, cells were stained with 1 μg/ml DAPI and observed with confocal laser scanning microscopy (LSM880, ZEISS).

### Immunoprecipitation (IP) assay

Briefly, HEK293T cells were transfected by p3*Flag-CMV-9-Myc plasmid following D347-2761 treatment for 48 h. Then the cells were lysed in NP-40 lysis buffer containing protease inhibitors. We added 30 μl anti-Flag agarose beads into cell extracts and incubated overnight at 4 °C. Then, the supernatant was discarded, beads were washed by rotating for 5 min at 4 °C with 500 μl TBS, and centrifuged at 2000 rpm for 3 min. The preceding steps were repeated three times. Finally, we suspended beads with 100 μl 2 * loading buffer and boiled for 10 min, subsequently analyzed by western blot.

### GST pull down assay

GST-tagged c-Myc protein and His-tagged Max protein were purchased from a commercial company (Proteintech). His-Max was incubated with GST-c-Myc and GST magnetic agarose (Sigma-Aldrich) for 3 h at 4 °C in 500 μl binding buffer (75 mM NaCl, 50 mM HEPES[pH 7.9]). Then a different dose of D347-2761 was added into the reaction system and incubated for another 2 h. Finally, the beads were washed 3 times, and c-Myc and Max protein were pulled down and detected by WB.

### Electrophoretic mobility shift assay (EMSA)

GST-c-Myc and His-Max protein have been mentioned in the above method. The experiment was conducted using LightShift Chemiluminescent EMSA Kit (Thermo, 20148) according to the manufacturer's protocol. Biotin 5′ end-labeled c-Myc response element (E-box): 5′-CCGGCTGACACGTGGTATTAATAG-3′, which contains c-Myc response element E-box motif.

### Q-PCR analysis

Total RNA was extracted by TRIzol reagent (Takara), and cDNA was synthesized using the M-MLV reverse transcriptase cDNA Synthesis Kit (Promega). Q-PCR experiment was conducted via Real-Time PCR System (Roche 480). The primers used in this study were listed in Additional file 1.

### Dual-luciferase reporter gene assay

The c-Myc regulatory element sequence was synthesized by GENEWIZ and cloned into the pGL4.20 vector. pcDNA3.1-c-Myc, pGL4.20-c-Myc-luc, and Renilla were transfected into HEK293T cells and subsequently treated with a given dose of compound D347-2761 for 24 h. Then the samples were prepared and carried out by Dual-Lumi™ II Luciferase Reporter Gene Assay Kit (Beyotime Biotechnology) according to the manufacturer’s protocol.

### Chromatin immunoprecipitation (ChIP)

Formaldehyde was added to cells treated or untreated by D347-2761 at a final concentration of 1% for 5 min at room temperature. The crosslinking was terminated by 125 mM Glycine and washed 3 times. Then cells were lysed by SDS lysis buffer and treated by an ultrasonic cell disruptor. Finally, samples were further processed using a ChIP assay kit (Millipore), and anti-c-Myc and IgG antibodies were used in this experiment, the final products were quantified by Q-PCR, and corresponding primers were listed in Additional file 1.

### Animal experiments

Briefly, 1 × 10^7^ RPMI-8226 cells were embedded subcutaneously in 6 week old NSG mice. When tumors were visible, the mice were randomly divided into two groups (n = 6) and treated with or without 35 mg/kg D347-2761 by intraperitoneal injection every other day for 2 weeks. The mouse weight and tumor volume were measured every 3 days. In the end, the mice were sacrificed, and tumors were fixed by 4% paraformaldehyde. Then c-Myc, PCNA (Proteintech), and Ki67 (Bioss) immunohistochemistry staining were performed. Subsequently, 5 × 10^6^ RPMI-8226-Luc cells were injected into the tail veins of NSG mice. 7 days later, mice were injected i.p. with 100 mg/kg D-luciferin (GoldBio) 5 min before in vivo imaging. When fluorescence signal was captured, the mice were randomly assigned into two groups and injected with or without 35 mg/kg D347-2761 every other day for 2 weeks, and the images were collected with PerkinElmer living imaging system. Finally, experimental mice's liver, spleen, and kidney tissues were removed and fixed, and the metastases were confirmed by HE staining. All experiments were approved by the Animal Care Committee of the Xuzhou Medical University, China.

### Statistical analysis

All data were presented as mean ± SD from at least three independent experiments. The students’ two-tailed *t* test was used to analyze the statistical difference. *P* < 0.05 was considered to be statistically significant. Statistical analyses were performed using GraphPad Prism 5 software.

## Results

### Molecular docking-based virtual screening and evaluation of candidate compounds

**Fig. 1:**
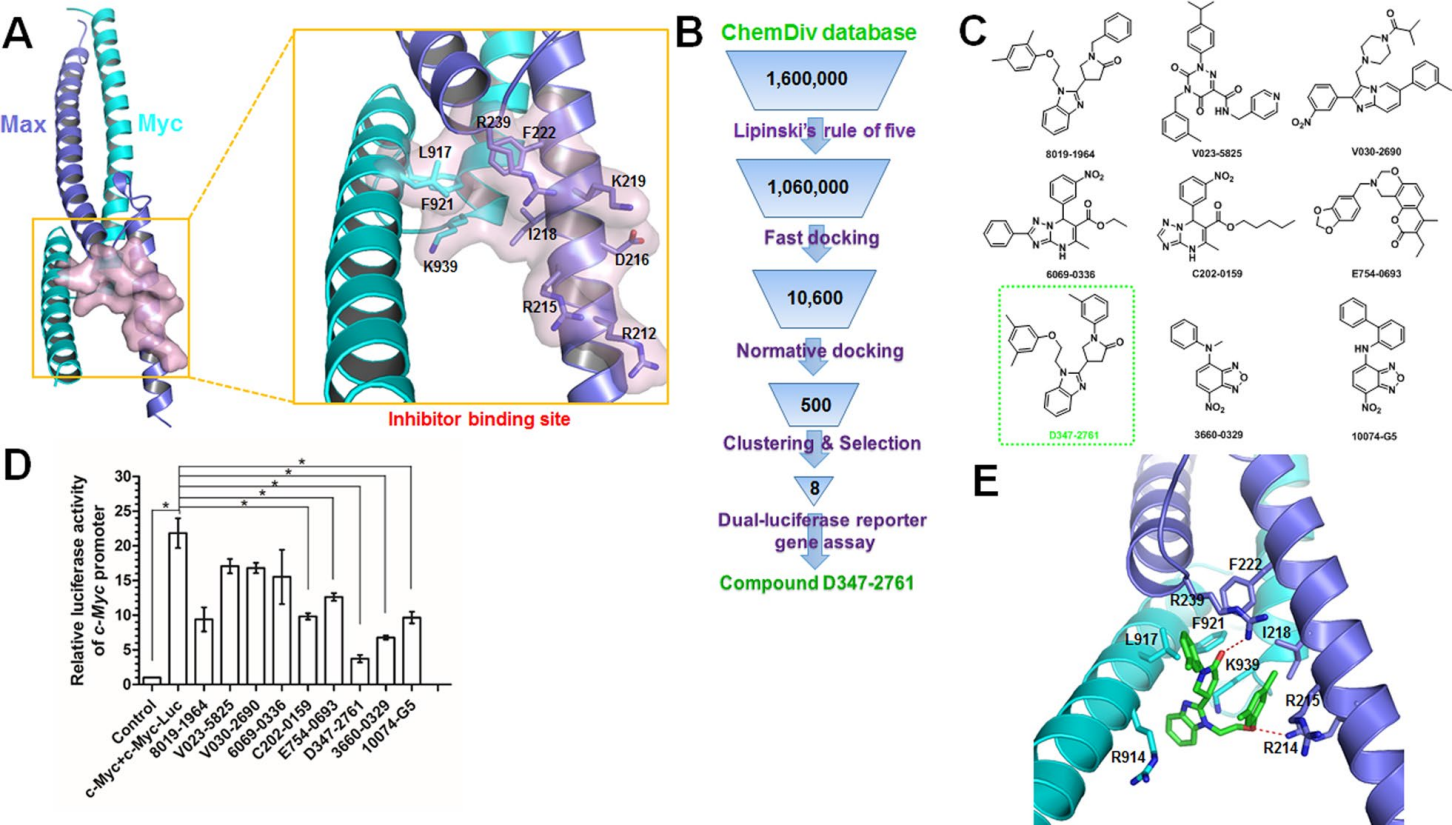
Molecular docking-based virtual screening for c-Myc inhibitors. **A** Crystal structure of c-Myc/Max complex and the well-defined inhibitor binding site formed by the interface of c-Myc/Max. Ten key residues attributed to the forming of the binding site were labeled. The proteins c-Myc and Max were shown in cartoon models and colored in cyan and blue, respectively. **B** Workflow of the molecular docking-based virtual screening. **C** 2D structures of eight hits from virtual screening and the control compound 10074-G5. **D** Dual-luciferase reporter gene assay for evaluating c-Myc transcriptional inhibitory activity in HEK293T cells treated by eight candidate compounds. Error bars: mean ± SD, *P < 0.05. **E** Molecular docking predicted binding mode of compound D347-2761. Compound D347-2761 and the key residues for its binding were shown in stick models. The hydrogen bond interaction was depicted in red dotted line

**Fig. 2:**
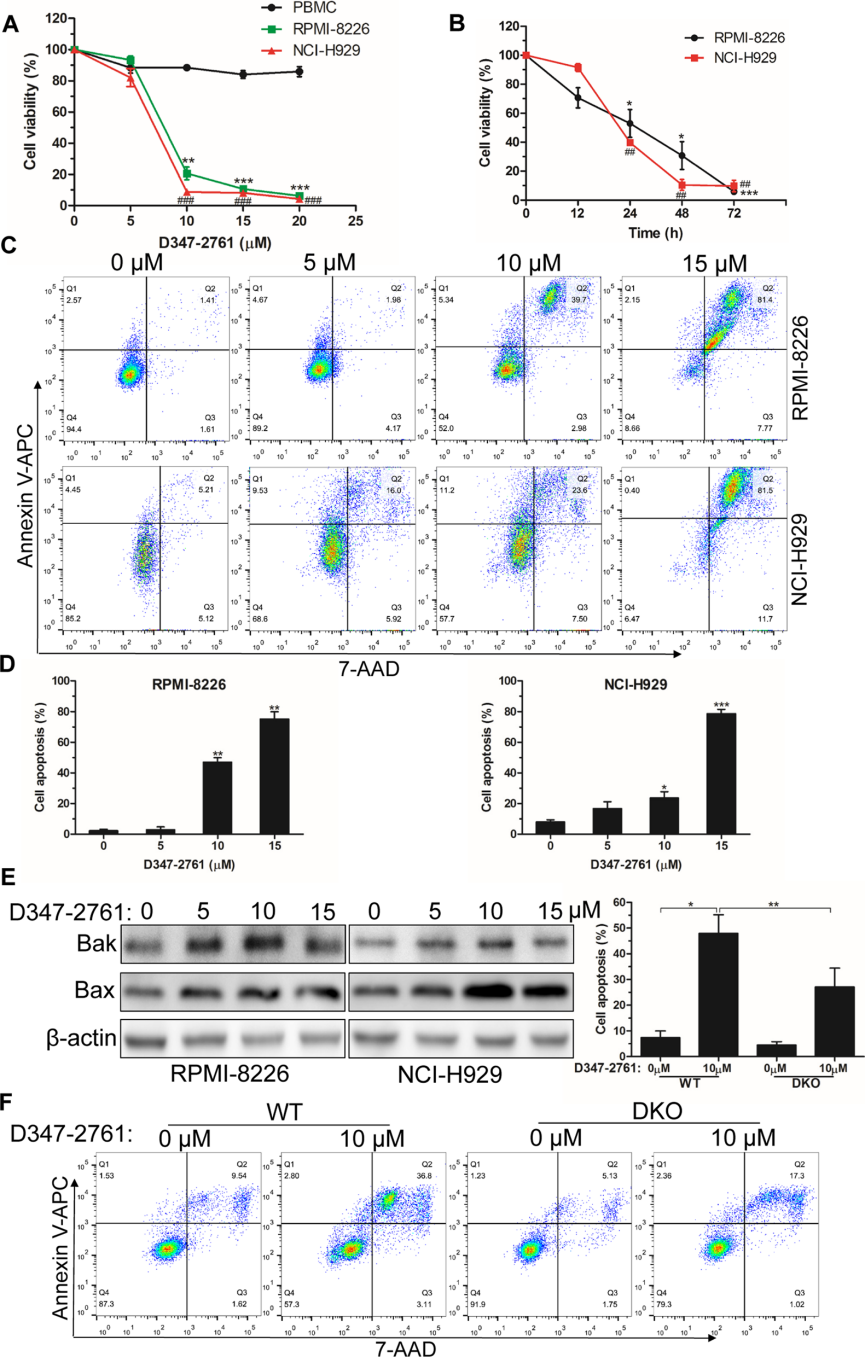
D347-2761 induced cytotoxicity in MM cells. **A** RPMI-8226 and NCI-H929 cells were treated by different dose of D347-2761 (5 μM, 10 μM, 15 μM and 20 μM) for 48 h and cell viabilities were measured using CCK8 kit. **B** Indicated cells treated by 10 μM D347-2761 at different time were dealt with CCK8 and cell survival rates were measured. **C**, **D** Flow cytometry analysis of apoptotic cell rates in RPMI-8226 and NCI-H929 cells by D347-2761 treatment (5 μM, 10 μM and 15 μM) for 48 h. **E** Western blot assay of Bak and Bax apoptotic markers in RPMI-8226 and NCI-H929 cells treated by D347-2761. β-actin was used to be internal control. **F** RPMI-8226-WT and –DKO cells were treated by 10 μM D347-2761 for 48 h and cell apoptosis was detected using Annexin V-APC/7-AAD apoptosis detection kit. Error bars: mean ± SD from at least three independent experiments. ^#^, *P < 0.05, ^##^, **P < 0.01, ^###^, ***P < 0.001

The well-defined binding pocket formed by the interface between c-Myc and Max was selected as the potential binding site of c-Myc inhibitors (Fig. 1A), and then the ChemDiv chemical library was virtually screened (Fig. 2B). Eight compounds (Fig. 1C) identified from the VS were tested for their c-Myc inhibitory activity, with 10074-G5 used as a positive control. The dual-luciferase reporter gene assay indicated that compound D347-2761 showed evident c-Myc transcriptional inhibitory activity at the concentration of 10 μM than others (Fig. 1D), which was selected as a potential inhibitor hit for further studies. According to the predicted binding mode of compound D347-2761 obtained from the above molecular docking-based virtual screening, it formed two hydrogen bonds with the side chains of Arg239 and Arg214 (Fig. 1E). Moreover, compound D347-2761 is fitted precisely in c-Myc/Max complex interface, with strong hydrophobic interactions to the residues Arg914, Leu917, Phe921, and Lys939 in c-Myc and Arg215, Ile218, and Phe222 in Max. In addition, the single-mutations of the above key residues to alaine via the same molecular docking studies revealed that the binding affinities of compound D347-2761 would decrease greatly, especially for the R239A and F921A mutants (Additional file 6: Table S1). Compound D347-2761 would form only one hydrogen bond with the main chain of Ala239 in the R239A mutant (Additional file 2: Figure S1A). Similar, the binding mode of D347-2761 would change greatly in the F921A mutant (Additional file 2: Figure S1B).

### Compound D347-2761 showed cytotoxicity for myeloma cells

As a potential c-Myc inhibitor, compound D347-2761 exhibited better transcriptional repressive activity than other compounds. Here, we found compound D347-2761 dose-and time-dependently inhibited RPMI-8226 and NCI-H929 cell viabilities significantly, but not normal PBMCs (Fig. 2A, B). Compared with commercial c-Myc inhibitor 10074-G5, D347-2761 showed lower cytotoxicity than 10,074-G5 (IC50:37.66 μM, Additional file 3: Figure S2A). Additionally, we silenced c-Myc expression in RPMI-8226 cells, and the results indicated that compound D347-2761 enhanced myeloma cell killing abilities in WT RPMI-8226 cells (Additional file 3: Figure S2B). Meanwhile, our EdU proliferation assay also showed that compound D347-2761 inhibited myeloma cell proliferation obviously (Additional file 3: Figure S2C–S2D). We then evaluated the effect of D347-2761 on MM cell apoptosis by flow cytometry, and the results demonstrated that D347-2761 induced RPMI-8226 and NCI-H929 cell apoptosis obviously in a concentration-dependent manner (Fig. 2C, D). Meanwhile, western blot analysis confirmed that D347-2761 could activate apoptosis-associated markers, including Bak and Bax (Fig. 2E), critical genes in the caspase-dependent endogenous apoptosis pathway, but not caspase 3, caspase 8 and caspase 9 (Additional file 4: Figure S3A). Subsequently, we knocked out the expression of Bak and Bax via CRISPR cas9 methods, and the silencing efficiency was measured at the mRNA and protein levels (Additional file 4: Figure S3B-S3C). Flow cytometry apoptosis assay indicated that dual knockout of Bak/Bax partially reversed compound D347-2761-mediated RPMI-8226 cell death (Fig. 2F). As a pan-caspase inhibitor, Z-VAD-FMK could not restore the D347-2761-induced apoptosis phenomenon (Additional file 4: Figure S3D), implying that compound D347-2761 led to myeloma cell apoptosis partly via a caspase-independent pathway. Moreover, we also found that D347-2761 did not change mitochondrial membrane potential (Additional file 4: Figure S3E), which was consistent with apoptosis experiments that D347-2761 mainly promoted cell late apoptosis.

### D347-2761 synergized with bortezomib to promote DNA damage

**Fig. 3:**
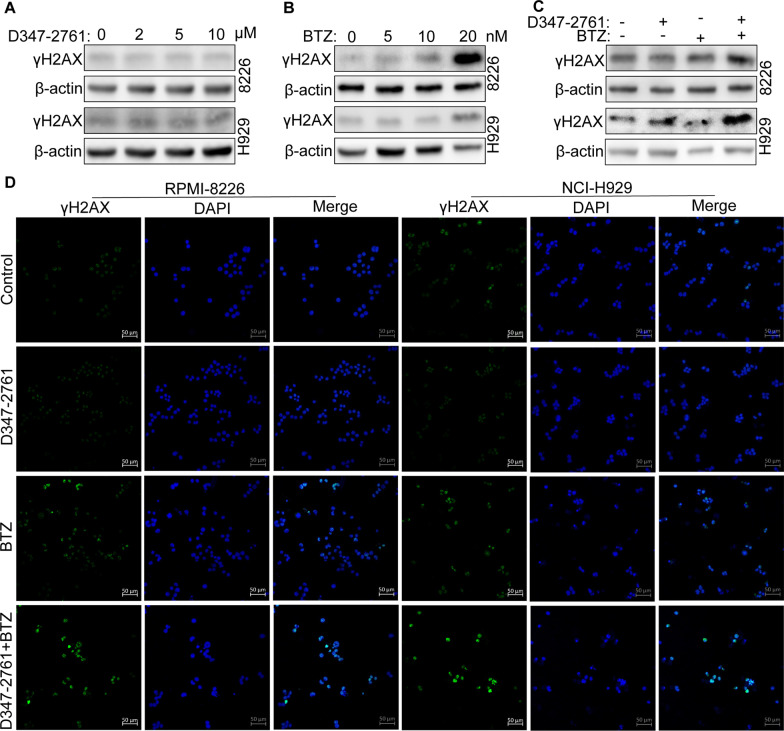
D347-2761 synergized with BTZ to promote DNA damage. **A** RPMI-8226 and NCI-H929 cells were treated by different dose of D347-2761 (2 μM, 5 μM and 10 μM) and western blot analysis of γH2AX expression. **B** Western bolt showing the expression of γH2AX in RPMI-8226 and NCI-H929 cells via different concentration of BTZ (5 nM, 10 nM and 20 nM). **C** RPMI-8226 and NCI-H929 cells were treated by 10 μM D347-2761 and/or 10 nM BTZ and western blot analysis of γH2AX expression. β-actin was used to be internal control. **D** Immunofluorescence assay of γH2AX expression level in indicated cells via treatment with 10 μM D347-2761 and/or 10 nM BTZ. The nucleus was stained by 1 μg/ml DAPI. Scale bars: 50 μm

Compound D347-2761 could lead to myeloma cell cycle S phase arrest in a dose-dependent manner (Additional file 5: Figure S4A). It has been proven that DNA damage repair was closely correlated with the cell cycle. Therefore, we detected whether D347-2761 induced DNA damage in MM cells. Surprisingly, γH2AX, an essential marker of DNA damage, was not activated in RPMI-8226 and NCI-H929 cells regardless of high or low concentration of D347-2761 treatment (Fig. 3A). On the contrary, as a first-line targeted therapeutic agent of MM approved by FDA, bortezomib (BTZ) dose-dependently promoted DNA damage via activating the expression of γH2AX in RPMI-8226 and NCI-H929 cells (Fig. 3B). We then estimated the impact of D347-2761 in combination with BTZ on DNA damage. As shown in Fig. 3C, compound D347-2761 could enhance the expression of γH2AX following treatment of BTZ. Additionally, immunofluorescence assay also confirmed that D347-2761 synergized with bortezomib to activate γH2AX to induce DNA damage in RPMI-8226 and NCI-H929 cells (Fig. 3D), suggesting that compound D347-2761 may play a crucial role in overcoming drug resistance.

### D347-2761 contributed to overcoming BTZ drug resistance in MM cells

**Fig. 4:**
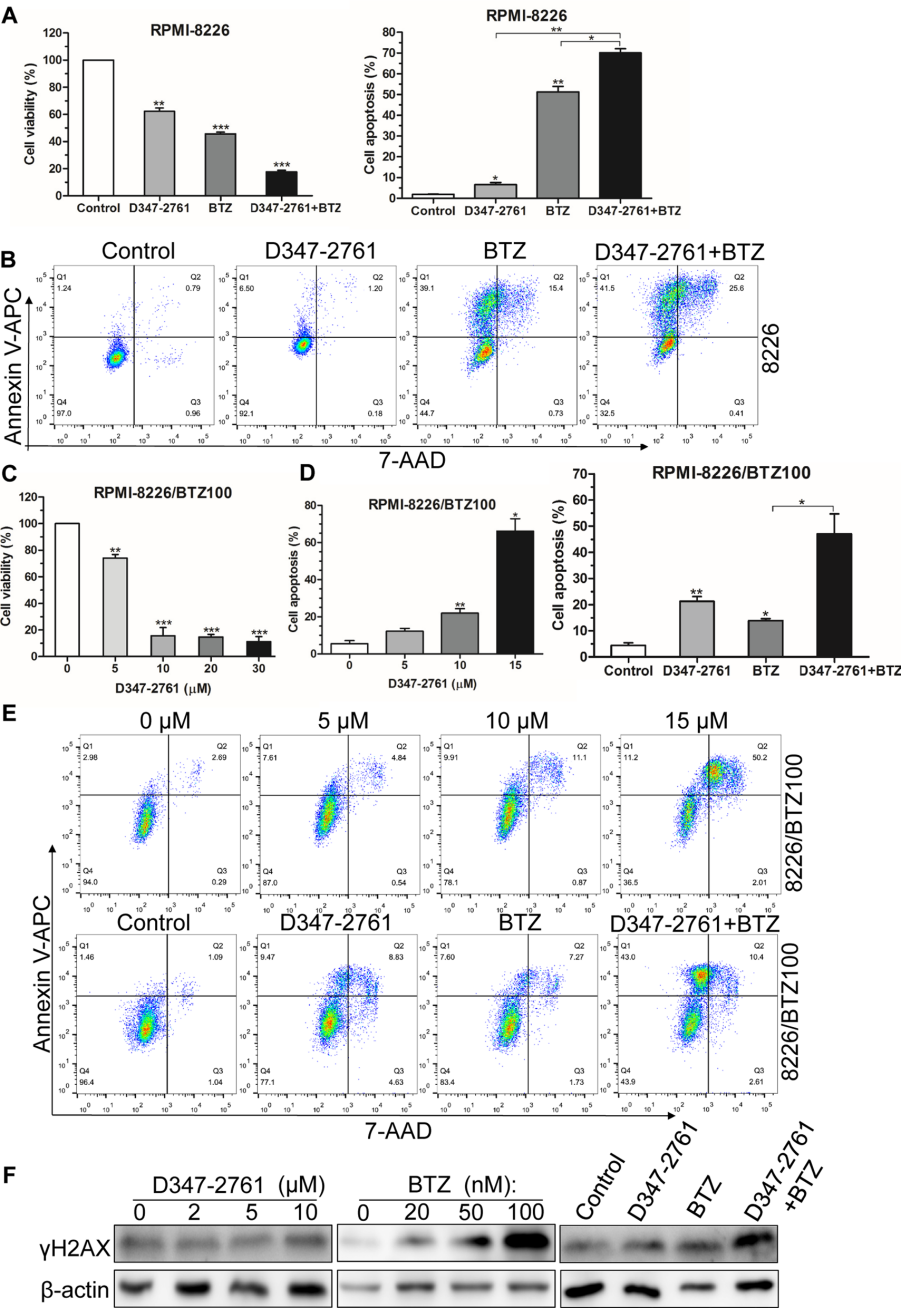
D347-2761 overcame BTZ drug resistance in MM cells. **A** RPMI-8226 cell was dealt with 5 μM D347-2761 following 10 nM BTZ treatment and cell viability was measured by CCK8 assay. **B** Flow cytometry assay analysis of apoptotic cell rate in RPMI-8226 cells following 5 μM D347-2761 and/or 10 nM BTZ treatment. **C** RPMI-8226/BTZ100 cell was treated by different dose of D347-2761 (5 μM, 10 μM, 20 μM and 30 μM) and cell viability was measured by CCK8 assay. D-E. Flow cytometry assay analysis of apoptotic cell rate in RPMI-8226/BTZ100 cells following different concentration of D347-2761 (5 μM, 10 μM and 15 μM) for 48 h, or treated by 5 μM D347-2761 and/or 10 nM BTZ treatment. **F** Western blot analysis of γH2AX expression in RPMI-8226/BTZ100 cells following different dose of D347-2761 or BTZ and combination (10 μM D347-2761 and 10 nM BTZ). β-actin was used to be internal control. Error bars: mean ± SD. *P < 0.05, **P < 0.01, ***P < 0.001

To explore the correlation of D347-2761 with drug resistance, we first evaluated the median dose effect using Chou-Talalay method, suggesting that D347-2761 exhibited a synergistic cytotoxicity with BTZ (CI < 1.0, Additional file 5: Figure S4B-S4C). CCK8 assay also showed that D347-2761 could further repress survival in combination with BTZ in RPMI-8226 cells (Fig. 4A). In the aspect of cell apoptosis, we found that D347-2761 synergized with BTZ to enhance apoptosis behavior in RPMI-8226 cells (Fig. 4B). Subsequently, we used 100 nM BTZ-resistant RPMI-8226 cells to evaluate the impact of D347-2761 on cell viability. As shown in Fig. 4C, D347-2761 dose-dependently inhibited RPMI-8226/BTZ100 cell proliferative ability. Annexin V-APC/7-AAD assay further indicated that compound D347-2761 facilitated RPMI-8226/BTZ100 cell death in a concentration-dependent manner and synergized with BTZ to promote apoptosis abilities in RPMI-8226/BTZ100 cells (Fig. D, E). It has been confirmed that D347-2761 could promote myeloma cell DNA damage via combining with BTZ. Herein, our results showed that D347-2761 did not activate the expression of γH2AX. Conversely, a high dose of BTZ still had a stimulation for DNA damage. Furthermore, the exciting finding was the presence of collaboration between D347-2761 and BTZ for enhancing DNA damage in RPMI-8226/BTZ100 cells (Fig. 4F). To sum it up, our preliminary studies demonstrated that compound D347-2761 contributed to overcoming bortezomib drug resistance in myeloma cells.

### D347-2761 inhibited myeloma growth and infiltration in vivo

**Fig. 5:**
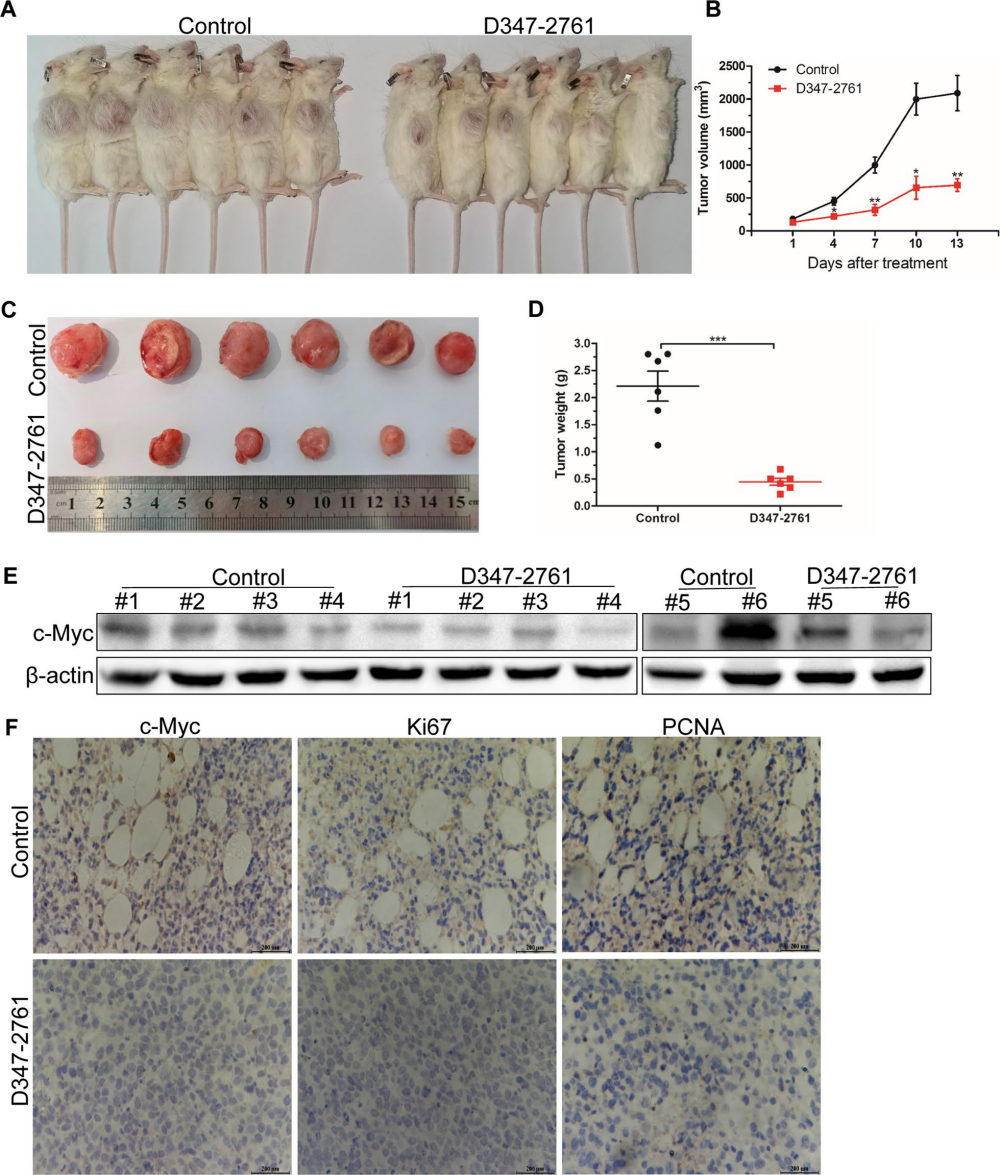
D347-2761 inhibited primary tumor growth in humanized myeloma xenograft mouse model. **A** NSG mice were separated with two groups: control group and D347-2761 group (n = 6). Tumor bearing status was shown by high digital camera. **B** Tumor volume of each group was measured every 3 days. **C** Tumors were stripped and tumor size of control group and D347-2761 group was shown respectively. **D** Statistical analysis of tumor weights of each group. **E** Western blot analysis of c-Myc expression in primary tumors of control and D347-2761 group. β-actin was used to be internal control. Error bars: mean ± SD. *P < 0.05, **P < 0.01, ***P < 0.001. **F** IHC staining analysis of c-Myc, Ki67 and PCNA in each group. Scale bars: 200 μm

**Fig. 6:**
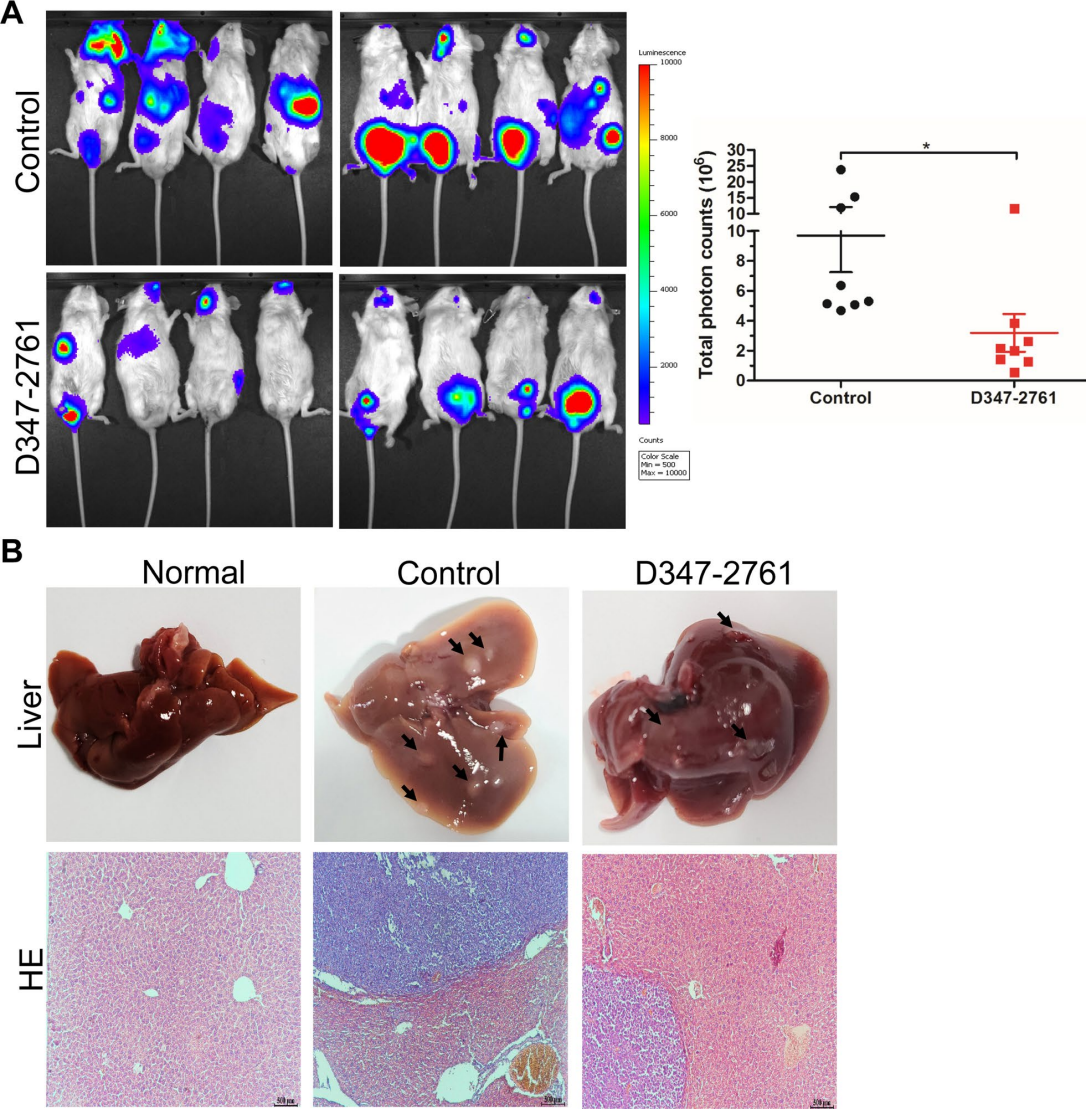
D347-2761 repressed myeloma distal infiltration. **A** NSG mice were injected with RPMI-8226-luc cells via tail vein and fluorescence signal of each group was captured using living imaging system. The total photon counts were also statistically analyzed. Error bars: mean ± SD. *P < 0.05. **B** Liver tissues of normal mice, control and D347-2761-treated group were shown, and HE staining analysis of metastases in each group. The arrow means micrometastasis foci

To evaluate the therapeutic potential of compound D347-2761 on MM disease, NSG mice were subcutaneously injected with RPMI-8226 cells and divided into two groups when tumors were visible. Then the mice were intraperitoneally administered with vehicle and D347-2761 every other day, respectively. Our results demonstrated that compound D347-2761 inhibited primary tumor growth significantly relative to the vehicle group (Fig. 5A, B). Subsequently, the tumors were stripped, and tumors weights of the D347-2761-treated group were smaller than the vehicle group (Fig. 5C, D). However, D347-2761 did not affect mice's weight compared with the vehicle group (data not shown). We then extracted 12 mice primary tumor proteins, and the western blot assay confirmed that c-Myc was significantly downregulated in the D347-2761-treated group compared with the control group (Fig. 5E). Additionally, immunohistochemical (IHC) staining further verified the significant downregulation of c-Myc, and D347-2761 decreased the expression of proliferative markers Ki67 and PCNA (Fig. 5F), which is consistent with the experiments in vitro. On the other hand, we also evaluated the impact of D347-2761 on myeloma distal infiltration. Luciferase-labeled RPMI-8226 cells were injected into the tail vein of NSG mice. Three weeks later, the fluorescence signal was captured using a living imaging system, and the results demonstrated that the vehicle group exhibited a more substantial capacity of distal infiltration than the D347-2761-treated group (Fig. 6A). Meanwhile, the mice were sacrificed, and the vital organs were stripped, including the liver, spleen, and kidney. Surprisingly, we only observed more and bigger liver metastases in the control group relative to the normal liver and D347-2761-treated group, which was consistent with the results of hematoxylin–eosin (HE) staining (Fig. 6B). Taken together, our studies in vitro and in vivo suggested that novel c-Myc inhibitor D347-2761 could be a potential therapeutic agent targeting multiple myeloma.

### Compound D347-2761 could block c-Myc/Max heterodimerization

**Fig. 7:**
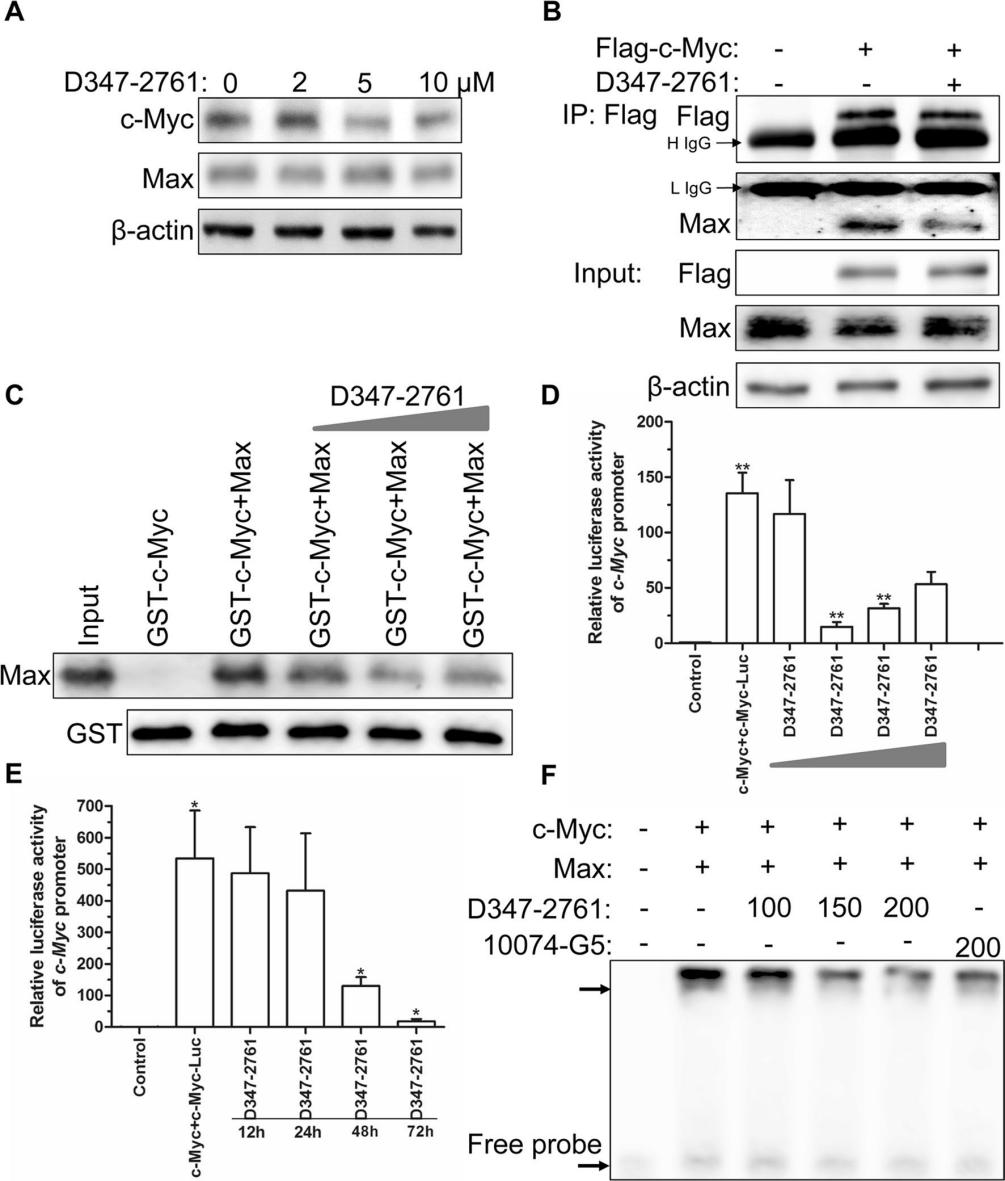
Compound D347-2761 interfered with c-Myc/Max heterodimerization. **A** Western blot analysis of c-Myc and Max expression in NCI-H929 cells treated by different dose of D347-2761 (2 μM, 5 μM and 10 μM). **B** HEK293T cells were transfected with 2 μg p3*Flag-CMV-9-c-Myc plasmids, then 10 μM D347-2761 was added into the medium for 48 h, IP assay analysis of c-Myc and Max expression. H IgG means heavy IgG and L IgG means light IgG. **C** GST pull down assay analysis of interaction between c-Myc and Max following different concentration of D347-2761 treatment. **D** HEK293T cells were transfected with c-Myc, c-Myc-luc and Renilla plasmids following different dose of D347-2761 treatment for 48 h, and luciferase reporter assay analysis of c-Myc promoter activity. **E** HEK293T cells were transfected with c-Myc, c-Myc-luc and Renilla plasmids following D347-2761 treatment for different time, and luciferase reporter assay analysis of c-Myc promoter activity. F. GST-c-Myc incubated with His-Max following different dose of D347-2761 treatment (100 μM, 150 μM and 200 μM), then biotin-labeled c-Myc response element probe was added into the reaction system for following EMSA assay

Theoretically, we have proven that compound D347-2761 could bind to the interface of c-Myc/Max via molecular docking-based virtual screening. Herein, we first evaluated the expression level of c-Myc and Max in NCI-H929 cells following treatment with different doses of D347-2761. The results showed that D347-2761 dose-dependently decreased the c-Myc expression, but not Max (Fig. 7A). Next, the Flag-tagged c-Myc plasmids were transiently transformed into HEK-293T cells following D347-2761 treatment for 48 h, and Flag affinity purification assay indicated that D347-2761 did not affect the expression of exogenous Flag-c-Myc and endogenous Max, but blocked the formation of c-Myc/Max heterodimer significantly (Fig. 7B). Furthermore, we purchased commercial GST-c-Myc and His-Max protein to conduct GST pull down assay, and the study demonstrated that the direct interaction between c-Myc and Max was decreased following treatment with an elevated dose of D347-2761 (Fig. 7C). The previous study has proven that c-Myc/Max heterodimer should bind to c-Myc response elements to further transcriptional activate downstream target genes. So we constructed a luciferase reporter gene plasmid based on the c-Myc response element E-box motif, as shown in Fig. 7D, E. Compound D347-2761 inhibited c-Myc-mediated transcriptional activities in a dose- and time-dependent manner. Subsequently, we further estimated whether D347-2761 directly interfered with c-Myc/Max complex binding to the E-box motif (CACGTG) probe. The EMSA assay showed that compound D347-2761 exhibited a dose-dependent inhibition of c-Myc/Max heterodimer (Fig. 7F). Thus, our studies confirmed that compound D347-2761 could significantly block c-Myc/Max heterodimerization.

### Compound D347-2761 disturbed c-Myc stability to regulate downstream target genes

**Fig. 8:**
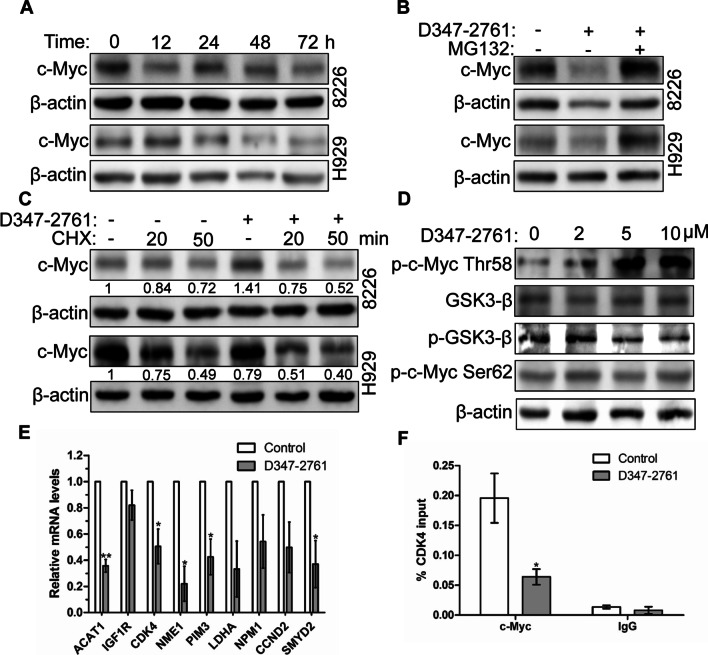
D347-2761 disturbed c-Myc stability to regulate downstream gene transcription. **A** Indicated cells were treated by 10 μM D347-2761 at different time (12 h, 24 h, 48 h and 72 h) and western blot analysis of c-Myc expression. **B** Indicated cells were treated by 10 μM D347-2761 following 10 μM MG132 treatment for 4 h, 24 h later, western blot assay of c-Myc expression. **C** RPMI-8226 and NCI-H929 cells were dealt with 10 μM D347-2761 or not for 6 h and then 5 μg/ml CHX were added into the medium for 20 min and 50 min respectively. Western blot anaylsis of c-Myc protein expression. **D** RPMI-8226 cells were treated by different dose of D347-2761 (2 μM, 5 μM and 10 μM) for 48 h, western blot analysis of p-c-Myc Thr58, p-c-Myc Ser62, p-GSK3-β and GSK3β. β-actin was used to be internal control. **E** Real time PCR analysis of potential c-Myc target genes mRNA levels in RPMI-8226 cells via D347-2761 treatment. **F** qChIP was performed to measure the levels of c-Myc at CDK4 promoter in RPMI-8226 cells after D347-2761 treatment. The experiments were repeated at least three times. Error bars: mean ± SD. *P < 0.05, **P < 0.01

Our preliminary studies have proven that compound D347-2761 did not downregulate the expression of c-Myc at the mRNA level (data not shown) but decreased the c-Myc protein expression in a time-dependent manner in RPMI-8226 and NCI-H929 cells (Fig. 8A). So we speculated that D347-2761 might disturb c-Myc protein stability to regulate its downstream target genes further. As shown in Fig. 8B, proteasome inhibitor MG132 could restore the expression of c-Myc in RPMI-8226 and NCI-H929 cells treated by D347-2761. Moreover, we used protein synthesis inhibitor cycloheximide (CHX) to detect the impact of D347-2761 on c-Myc protein half-life, and the results showed that D347-2761 inhibited the synthesis of c-Myc via shortening its half-life in myeloma cells (Fig. 8C). Many studies have confirmed that the phosphorylation of c-Myc Thr58 promoted its protein degradation. Conversely, elevating the c-Myc Ser62 phosphorylation level enhanced its protein stability. Our studies demonstrated that compound D347-2761 dose-dependently increased the phosphorylation level of c-Myc Thr58 and decreased GSK3β phosphorylation level but had no impact on GSK3β and c-Myc Ser62 phosphorylation (Fig. 8D), implying that D347-2761 disturbed c-Myc protein stability mainly via upregulating its Thr58 phosphorylation level. To further elucidate the function of c-Myc in tumor progression, scientists have been focusing on identifying c-Myc target genes and their role in c-Myc–mediated physiological and pathological events. Zeller et al. [28] delineated a c-Myc transcriptional network based on ChIP coupled with pair-end ditag sequencing analysis (ChIP-PET) and found 668 direct c-Myc-regulated target genes. Then we screened nine genes containing c-Myc response elements E-box (CACGTG), including ACAT1, IGF1R, CDK4, NME1, PIM3, LDHA, NPM1, CCND2, and SMYD2. As shown in Fig. 8E, compound D347-2761 inhibited the expression of ACAT1, CDK4, NME1, PIM3, and SMYD2 at the mRNA levels in RPMI-8226 cells. Meanwhile, we detected the binding of c-Myc protein on ACAT1, CDK4, NME1, PIM3, and SMYD2 gene promoters using ChIP methods. The results indicated that compound D347-2761 decreased c-Myc on CDK4 promoter, but not others (Fig. 8F). Thus, compound D347-2761 could disturb c-Myc stability to reduce its downstream CDK4 gene expression.

## Discussion

It has been proven that c-Myc gene rearrangement played a vital role in the pathogenesis of plasma cell disease. Moreover, c-Myc expression was also involved in the progress from monoclonal gammopathy of unknown significance and smoldering multiple myeloma (MM) to MM and plasma cell leukemia [29, 30]. More and more evidence indicated that c-Myc mainly regulated cell viabilities through activating cell cycle proteins, cell differentiation, angiogenesis, increasing genome instability, and inducing tumorigenesis [31]. Therefore, targeting c-Myc may be an effective strategy for MM therapy in the future. In 2011, Delmore et al. confirmed that bromodomain and extra-terminal (BET) inhibition was performed as a therapeutic method to target c-Myc. BET inhibitor JQ1 could downregulate c-Myc in a time-dependent manner in MM cells, and the MM xenograft model treated by JQ1 also significantly decreased MM tumor burden and increased overall survival [9]. Currently, some BET inhibitors such as OTX015 and CPI-0610 exhibited well anti-myeloma activities in vitro and in vivo, which had been approved to conduct clinical I phase studies (NCT02157636, NCT 01713582) [32, 33]. Herein, we screened and identified a novel c-Myc inhibitor D347-2761, which could simultaneously target c-Myc/Max heterodimer and c-Myc unstable domain. Our studies confirmed that D347-2761 dose-dependently inhibited myeloma cell proliferation and induced cell apoptosis process via activating Bak and Bax. Nevertheless, pan-caspase inhibitor Z-VAD-FMK did not restore D347-2761-mediated cell apoptosis behavior, suggesting that D347-2761 promoted myeloma cell death through caspase-dependent or independent pro-apoptotic pathway. On the other hand, we further knocked out the expression of Bak and Bax in RPMI-8226 cells. Excitingly, dual knockout of Bak/Bax partially decreased D347-2761-induced cell apoptosis, implying that compound D347-2761 could partly stimulate the endogenous apoptosis process.

As a malignant hematological disease, MM remains incurable, mainly due to drug-resistant problems. In this study, we found D347-2761 repressed BTZ-resistant myeloma cell proliferation and induced its apoptosis in a concentration-dependent manner, which manifested that D347-2761 overcame BTZ drug resistance to some extent. Additionally, our results also showed that D347-2761 synergized with BTZ to enhance its pro-apoptotic abilities. Genomic instability frequently occurs in MM, which is a prominent feature of MM cells [34, 35]. Herein, we found that D347-2761 did not induce myeloma cell DNA damage singly. Conversely, D347-2761 could, in combination with BTZ, increase the expression of γH2AX to result in DNA damage. Once DNA damage occurs, DNA double-strand breaks (DSBs) need to be repaired via two pathways: non-homologous end joining (NHEJ) and homologous recombination (HR) [36]. Therefore, our following work will explore the possible mechanism of D347-2761 combining with BTZ as DSBs repair inhibitors, which would suggest potential therapeutic opportunities for refractory and relapsed MM.

c-Myc protein stability plays an important role in regulating tumor progression. Moreover, our studies demonstrated that compound D347-2761 disturbed its stability in a time-dependent manner via activating the phosphorylation level of c-Myc Thr58, which resulted in c-Myc protein instability and degradation. GSK3β was the only known kinase to phosphorylate c-Myc Thr58, but it was not changed obviously in RPMI-8226 cells treated by D347-2761 (Fig. 8D). Generally, GSK3β accumulated in the cytoplasm in inactive form owing to AKT1-mediated phosphorylation. In embryonic stem cell self-renewal, GSK3β shuttled from cytoplasm to nucleus to targets c-Myc through phosphorylation on Thr58 [37]. Surprisingly, our immunofluorescence assay indicated that D347-2761 did not increase the distribution of GSK3β in the nucleus (data not shown). Likewise, we further detected the expression of GSK3β and its subcellular localization in D347-2761-treated RPMI-8226 cells following treatment with GSK3β kinase inhibitor LiCl, and the results showed that the phosphorylation of c-Myc Thr58 was still activated despite inhibition of GSK3β activity (data not shown). BRD4 is a transcriptional regulator participating in many cellular functions, such as transcription, DNA damage repair [38]. BRD4 also contributes to reactivate the transcription of c-Myc at the end of mitosis [9]. Another study suggested that BRD4 could interact with c-Myc [39], implying that BRD4 could maintain the homeostasis of c-Myc. Recently, Devaiah et al. [40] confirmed that BRD4 could phosphorylate c-Myc Thr58 further to promote its ubiquitination and degradation. In our study, whether compound D347-2761 interfered with the expression of BRD4 or hindered the interaction between c-Myc and BRD4 was worthy of exploring in depth.

To further delineate the possible c-Myc transcriptional target, q-PCR was used to analyze the expression levels of genes containing c-Myc response elements E-box including ACAT1, IGF1R, CDK4, NME1, PIM3, LDHA, NPM1, CCND2, and SMYD2. Our results showed that compound D347-2761 inhibited the transcription of ACAT1, CDK4, NME1, PIM3, and SMYD2 in RPMI-8226 cells. Our ChIP assay further detected a significant decrease of c-Myc on CDK4 promoter in D347-2761-treated cells, consistent with a previous report that c-Myc increased CDK4 mRNA levels through highly conserved c-Myc binding sites (E-box) within CDK4 promoter [41]. Our studies identified a novel dual-targeting c-Myc inhibitor D347-2761, which could block c-Myc/Max heterodimerization and disturb c-Myc protein stability to further repress multiple myeloma growth in vitro and in vivo via inhibiting CDK4 promoter transcriptional activity.

## Conclusions

We identify a novel dual-targeting c-Myc inhibitor D347-2761 targeting c-Myc unstable domain and c-Myc/Max heterodimerization, which further inhibits myeloma growth and infiltration in vitro and in vivo via regulating downstream CDK4 promoter transcriptional activity. This study suggests compound D347-2761 could be a potential therapeutic agent for multiple myeloma.

## Supplementary Information

**Additional file 1:** The primer sequences of Q-PCR and ChIP.

**Additional file 2:** **Figure S1.** Binding modes of compound D347-2761 to the R239A (A) and F921A (B) c-Myc/Max mutants obtained from molecular docking studies.

**Additional file 3:** **Figure S2.** A. RPMI-8226 cells were treated by different dose of 10074-G5 (10μM, 20μM, 30μM, 40μM and 50μM) for 48h and cell viabilities were measured using CCK8 kit. B. RPMI-8226-shCtrl and shMyc#1-#4 cells were treated by different dose of D347-2761 for 48h and cell viabilities were measured using CCK8 kit. C-D. EdU incorporation assay, in which the viability of RPMI-8226 and NCI-H929 cells treated by 10μM D347-2761 for 48h was assessed based on immunofluorescence. Scale bars: 50μm. Error bars: mean ± SD. *P < 0.05, **P < 0.01, ***P<0.001.

**Additional file 4:** **Figure S3.** A. Western blot analysis of expression of caspase 3, caspase 8 and caspase 9 in RPMI-8226 cells treated by different concentration of D347-2761. B. Real-time PCR analysis of mRNA level of Bak and Bax in RPMI-8226-WT and -DKO cell lines. C. Western blot analysis of expression of Bak and Bax in RPMI-8226-WT and -DKO cell lines. β-actin was used to be internal control. D. Flow cytometry analysis of cell apoptosis following treatment with D347-2761 and/or Z-VAD-FMK in RPMI-8226 and NCI-H929 cells. E. Flow cytometry analysis of apoptosis and mitochondrial membrane potential in RPMI-8226 cells treated by 10μM D347-2761 for different time (12h, 24h and 48h). Error bars: mean ± SD, **P < 0.01, ***P < 0.001.

**Additional file 5:** **Figure S4.** A. Indicated cells were treated by 5μM and 10μM D347-2761for 48h, and the cell cycle was measured by flow cytometry. The ratio of cell cycle phase was statistically analyzed. B-C. Median dose effect analysis of synergistic anti-myeloma function of D347-2761 and BTZ in RPMI-8226 and RPMI-8226/BTZ100 cells. Combination index (CI) < 1 refers to synergy. Error bars: mean ± SD, *P < 0.05, **P < 0.01.

**Additional file 6: Table S1.** The docking scores of all mutations.

## Data Availability

Supporting data were available to all researchers.

## Abbreviations

MM: Multiple myeloma
GST: Glutathione S-transferase
BTZ: Bortezomib
CDK4: Cyclin-dependent kinase 4
bHLH-LZ: Basic helix-loop-helix leucine zipper
GSEA: Gene set enrichment analysis
MGUS: Monoclonal gammopathy of undetermined significance
WB: Western blot
IF: Immunofluorescence
IP: Immunoprecipitation
EMSA: Electrophoretic mobility shift assay
Q-PCR: Quantitative polymerase chain reaction
ChIP: Chromatin immunoprecipitation
CHX: Cycloheximide
IHC: Immunohistochemical
HE: Hematoxylin–eosin
