# Supplementary material for: Novel dual-targeting c-Myc inhibitor D347-2761 represses myeloma growth via blocking c-Myc/Max heterodimerization and disturbing its stability
Source: Cell Commun Signal. 2022 May 26;20:73. doi: 10.1186/s12964-022-00868-6 (PMC9137135; doi:10.1186/s12964-022-00868-6)
Supplement: Supplementary file 2 — Additional file 1: The primer sequences of Q-PCR and ChIP. [file 12964_2022_868_MOESM2_ESM.docx]

**Supplementary materials:**

**pGL4.20-c-Myc-luc vector construction:**

The c-Myc regulatory element sequence was synthesized by GENEWIZ including two restriction enzymes (XhoI, EcoRV): CTCGAGGGCCTAACTGGCCGGTACCGCTAGCCTCGATCACGTGCACGTGCACGTGCACGTGGCGCGTAGATCTGCAGAAGCTTAGACACTAGAGGGTATATAATGGGATATC, E-box motif (CACGTG) were highlighted. The pGL4.20 vector was cut using XhoI and EcoRV restriction enzymes, then the fragment and pGL4.20 vector were connected using T4 DNA Ligase and corresponding buffer. The products were transformed into DH5α competent cells, coated plates and selected monoclonal colony PCR to obtain correct pGL4.20-c-Myc-luc vector for sequencing and experiment.

**RT-PCR primers sequences:**

c-Myc:

Sense: ACCACCAGCAGCGACTCTGA

Antisense: TCCAGCAGAAGGTGATCCAGACT

ACTA1:

Sense: GGAGGCTGGTGCAGGAAATA

Antisense: TGCCTTTTCAATGGCTCCCT

IGF1R:

Sense: AAGGAATGAAGTCTGGCTCCG

Antisense: CCGCAGATTTCTCCACTCGT

CDK4:

Sense: GTGAGGGTCTCCCTTGATCTG

Sense: GAGACCAACCCTGCAGACTC

Antisense: GATCACGGGCCTTGTACACT

NME1:

Antisense:GGTCTGCCCTCCTGTCATTC

PIM3:

Sense: CGCAGGACCTCTTCGACTTTA

Antisense:CGCAGGTCCACAAGCAGATT

LDHA:

Sense: ACGTGCATTCCCGATTCCTT

Antisense:AACAGCACCAACCCCAACAA

NPM1:

Sense: CCAGCCAAAAATGCACAAAAGT

Antisense:GTAGTGCCCAGGACTGTTCAA

Sense: ACTGCGAGTACTGCTTCACC

CCND2:

Sense: AGAAGCTGTCTCTGATCCGC

Antisense:GCTCAGTCAGGGCATCACAA

SMYD2:

Antisense:TGTGCATGGGCCAATCTTCT

Actin:

Sense: TCGTGCGTGACATTAAGGAG Antisense:ATGCCAGGGTACATGGTGGT

**ChIP primers sequences:**

CDK4:

Sense: AGAGCAATGTCAAGCGGTCA

Antisense: GACAGGAGGTGCTTCGACTG

ACAT1:

Sense: TGAGTTTGTGCTGGGTAGGC

Antisense: TTGACGAGTGAAGGACGTGG

NME1:

Sense: GTTCTGCAAAATGGGCTCTCC

Antisense: CCCGAACACCTCTTACCCTT

PIM3:

Sense: AGGGTGAGGAGGAAGCATCT

Antisense: GGCCTCGAGGTGACACAC

SMYD2:

Sense: CGTTTCAGGCTTTGTGGGCT

Antisense: AGGCTGTTGGGGGATAATGC
