# Supplementary figures and images for: Novel dual-targeting c-Myc inhibitor D347-2761 represses myeloma growth via blocking c-Myc/Max heterodimerization and disturbing its stability
Source: Cell Commun Signal. 2022 May 26;20:73. doi: 10.1186/s12964-022-00868-6 (PMC9137135; doi:10.1186/s12964-022-00868-6)

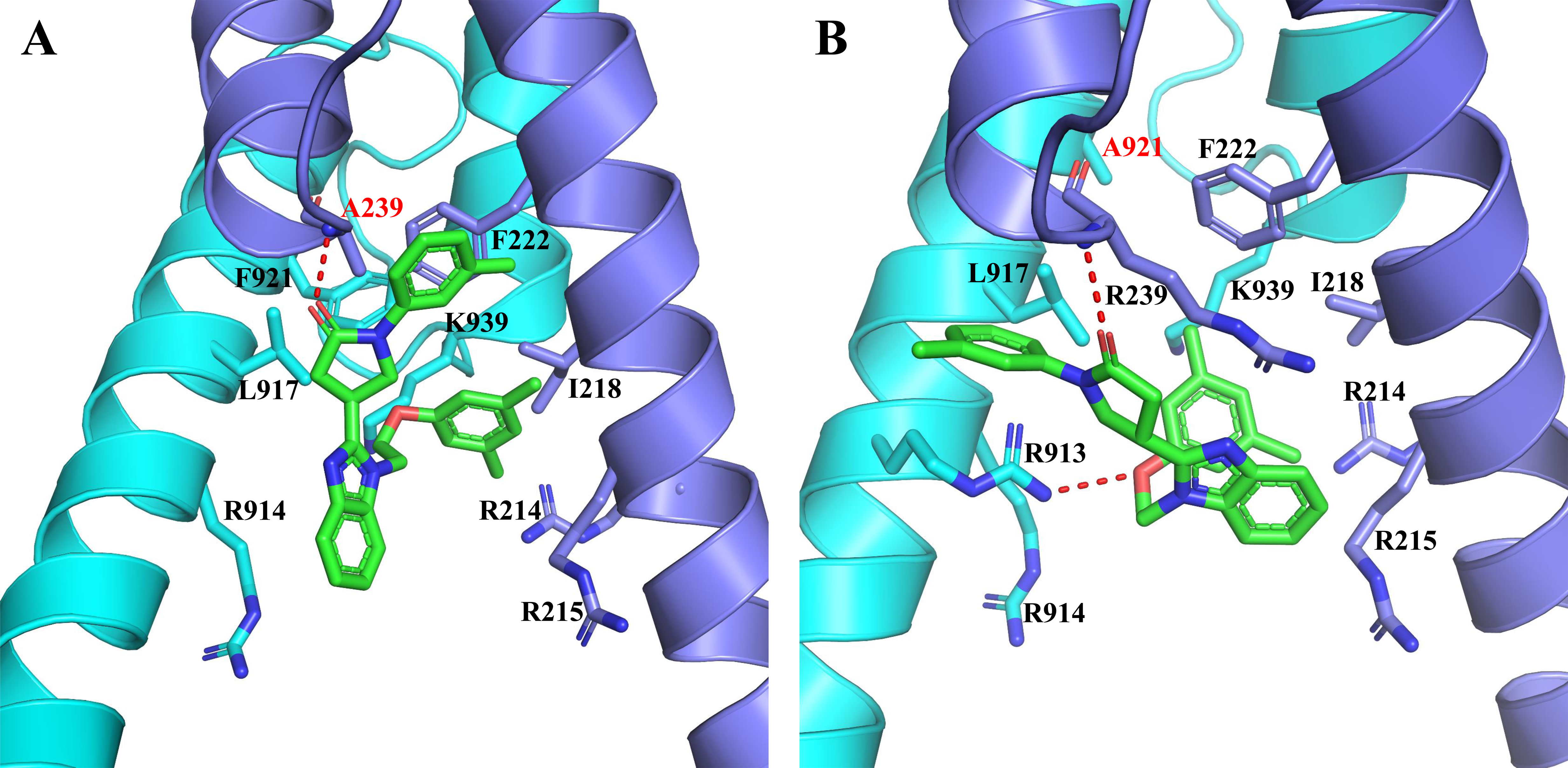

Supplement: Supplementary file 3 — Additional file 2: Figure S1. Binding modes of compound D347-2761 to the R239A (A) and F921A (B) c-Myc/Max mutants obtained from molecular docking studies. [file 12964_2022_868_MOESM3_ESM.tif]

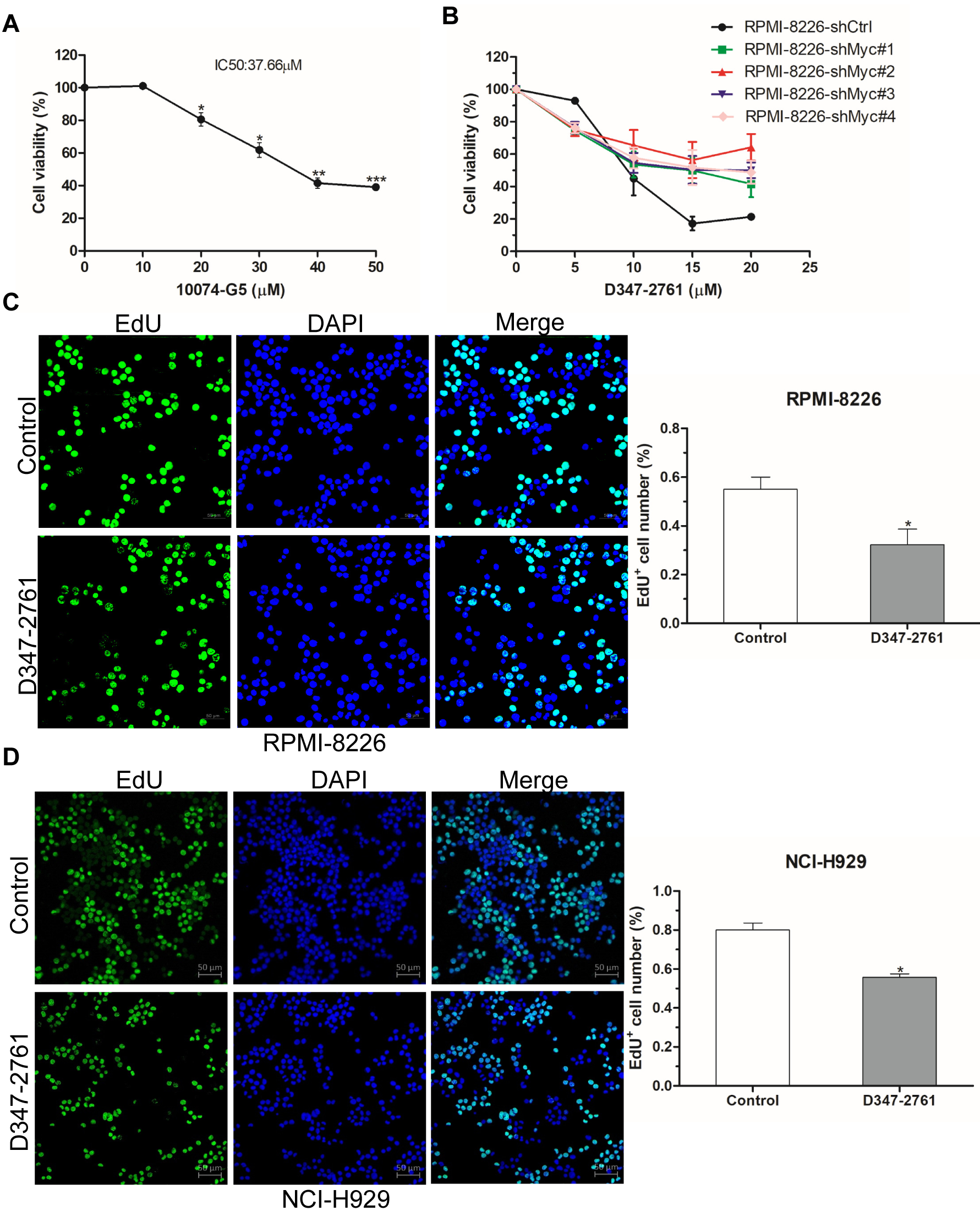

Supplement: Supplementary file 4 — Additional file 3: Figure S2. A. RPMI-8226 cells were treated by different dose of 10074-G5 (10μM, 20μM, 30μM, 40μM and 50μM) for 48h and cell viabilities were measured using CCK8 kit. B. RPMI-8226-shCtrl and shMyc#1-#4 cells were treated by different dose of D347-2761 for 48h and cell viabilities were measured using CCK8 kit. C-D. EdU incorporation assay, in which the viability of RPMI-8226 and NCI-H929 cells treated by 10μM D347-2761 for 48h was assessed based on immunofluorescence. Scale bars: 50μm. Error bars: mean ± SD. *P < 0.05, **P < 0.01, ***P<0.001. [file 12964_2022_868_MOESM4_ESM.tif]

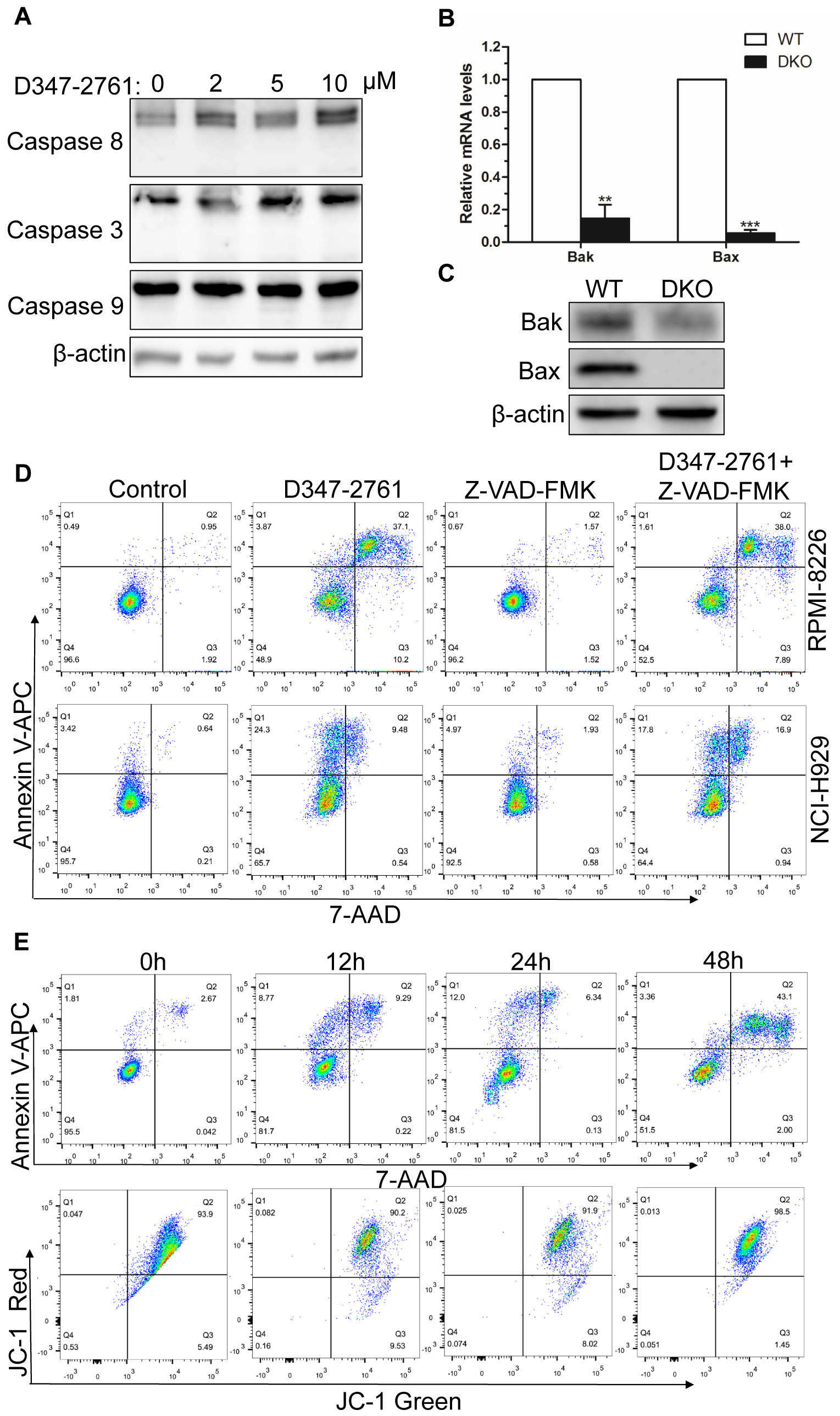

Supplement: Supplementary file 5 — Additional file 4: Figure S3. A. Western blot analysis of expression of caspase 3, caspase 8 and caspase 9 in RPMI-8226 cells treated by different concentration of D347-2761. B. Real-time PCR analysis of mRNA level of Bak and Bax in RPMI-8226-WT and -DKO cell lines. C. Western blot analysis of expression of Bak and Bax in RPMI-8226-WT and -DKO cell lines. β-actin was used to be internal control. D. Flow cytometry analysis of cell apoptosis following treatment with D347-2761 and/or Z-VAD-FMK in RPMI-8226 and NCI-H929 cells. E. Flow cytometry analysis of apoptosis and mitochondrial membrane potential in RPMI-8226 cells treated by 10μM D347-2761 for different time (12h, 24h and 48h). Error bars: mean ± SD, **P < 0.01, ***P < 0.001. [file 12964_2022_868_MOESM5_ESM.tif]

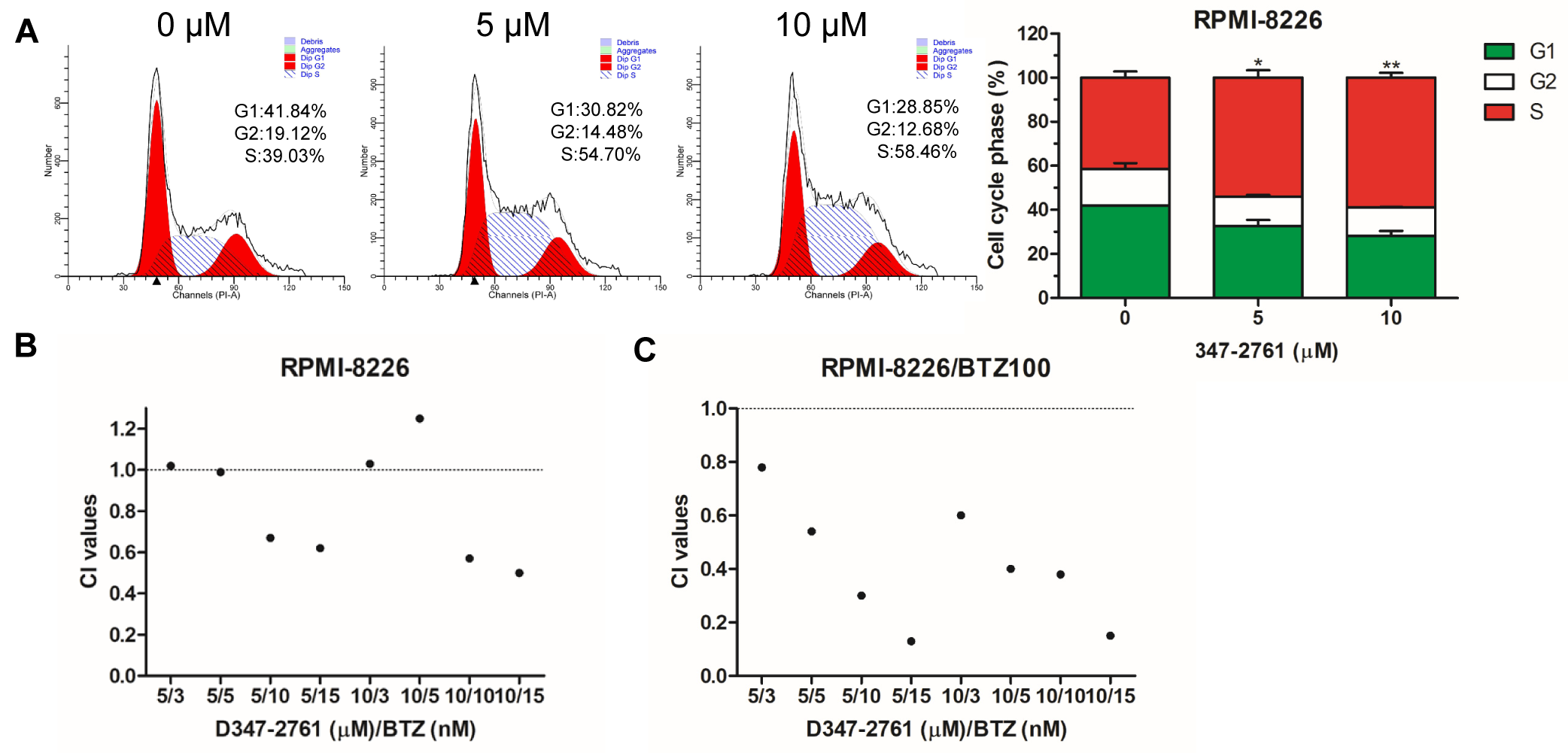

Supplement: Supplementary file 6 — Additional file 5: Figure S4. A. Indicated cells were treated by 5μM and 10μM D347-2761for 48h, and the cell cycle was measured by flow cytometry. The ratio of cell cycle phase was statistically analyzed. B-C. Median dose effect analysis of synergistic anti-myeloma function of D347-2761 and BTZ in RPMI-8226 and RPMI-8226/BTZ100 cells. Combination index (CI) < 1 refers to synergy. Error bars: mean ± SD, *P < 0.05, **P < 0.01. [file 12964_2022_868_MOESM6_ESM.tif]
