# Supplementary material for: Novel dual-targeting c-Myc inhibitor D347-2761 represses myeloma growth via blocking c-Myc/Max heterodimerization and disturbing its stability
Source: Cell Commun Signal. 2022 May 26;20:73. doi: 10.1186/s12964-022-00868-6 (PMC9137135; doi:10.1186/s12964-022-00868-6)
Supplement: Supplementary file 7 — Additional file 6: Table S1. The docking scores of all mutations. [file 12964_2022_868_MOESM7_ESM.docx]

**Table S1** Docking scores (-log(K_d_)) of compound D347-2761 to wild type c-Myc/Max or single-mutated proteins obtained from molecular docking studies.

|  | Wild type | R214A | R215A | I218A | F222A | R239A | R914A | L917A | F921A | K939A |
| --- | --- | --- | --- | --- | --- | --- | --- | --- | --- | --- |
| Docking  Score | 7.04 | 6.41 | 6.67 | 6.74 | 6.41 | 6.06 | 6.47 | 6.53 | 6.37 | 6.56 |
